# Genetic Evidence of Tiger Population Structure and Migration within an Isolated and Fragmented Landscape in Northwest India

**DOI:** 10.1371/journal.pone.0029827

**Published:** 2012-01-11

**Authors:** Patlolla Anuradha Reddy, Digpal Singh Gour, Maradani Bhavanishankar, Kanika Jaggi, Shaik Mohammed Hussain, Katakam Harika, Sisinthy Shivaji

**Affiliations:** Centre for Cellular and Molecular Biology, Hyderabad, India; Lund University, Sweden

## Abstract

**Background:**

Majority of the tiger habitat in Indian subcontinent lies within high human density landscapes and is highly sensitive to surrounding pressures. These forests are unable to sustain healthy tiger populations within a tiger-hostile matrix, despite considerable conservation efforts. Ranthambore Tiger Reserve (RTR) in Northwest India is one such isolated forest which is rapidly losing its links with other tiger territories in the Central Indian landscape. Non-invasive genetic sampling for individual identification is a potent technique to understand the relationships between threatened tiger populations in degraded habitats. This study is an attempt to establish tiger movement across a fragmented landscape between RTR and its neighboring forests, Kuno-Palpur Wildlife Sanctuary (KPWLS) and Madhav National Park (MNP) based on non-invasively obtained genetic data.

**Methods:**

Data from twelve microsatellite loci was used to define population structure and also to identify first generation migrants and admixed individuals in the above forests.

**Results:**

Population structure was consistent with the Central Indian landscape and we could determine significant gene flow between RTR and MNP. We could identify individuals of admixed ancestry in both these forests, as well as first generation migrants from RTR to KPWLS and MNP.

**Conclusions:**

Our results indicate reproductive mixing between animals of RTR and MNP in the recent past and migration of animals even today, despite fragmentation and poaching risk, from RTR towards MNP. Substantial conservation efforts should be made to maintain connectivity between these two subpopulations and also higher protection status should be conferred on Madhav National Park.

## Introduction

Despite tremendous pressures of an ever-exploding human population, India ranks eighth among the world's seventeen megabiodiversity countries [1]. Many communities in India live in abject poverty and depend heavily on forests for their livelihood. Agriculture and various commercial/industrial activities place further pressure on India's priceless ecosystems. The fate of the tiger, a large predator at the head of the food chain, is a good indicator of the conservation status of India's natural habitats and wildlife [2]–[4]. Project Tiger, initiated in 1973, envisioned the protection and management of high priority national parks, sanctuaries and surrounding reserve forests as tiger reserves. These tiger reserves initially garnered considerable attention and resources for tiger conservation, but as time passed and human populations increased, resources outside the protected areas were destroyed, increasing pressure on the protected areas and conflict with their wildlife. Ranganathan *et al.* [5] developed a landscape scale, density-based model to assess the impact of the surrounding landscape on the future survival of tigers in 150 reserves in the Indian subcontinent. Their findings highlighted that only 21 prime tiger reserves were relatively insensitive to the surrounding matrix. The remaining majority of the protected areas were highly sensitive to surrounding pressures, and were unable to sustain healthy tiger populations within a tiger-hostile matrix, despite considerable conservation efforts. Tigers in such vulnerable protected areas can only persist as part of larger populations that extend into surrounding forests.

**Figure 1:**
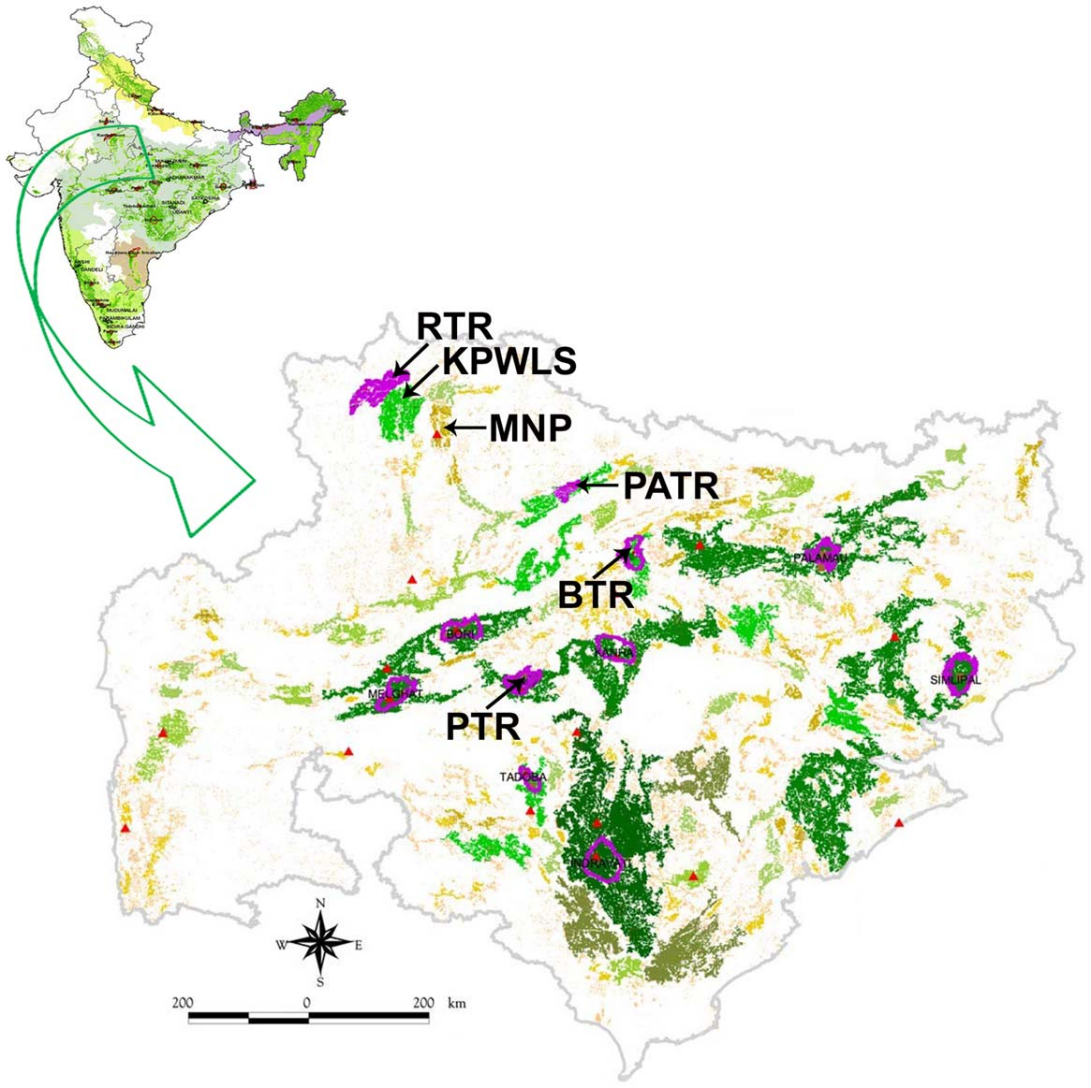
Map showing locations of the forests in Central Indian Landscape discussed in this study (modified from Jhala *et al.*, [**42**]). RTR – Ranthambore Tiger Reserve, KPWLS – Kuno-Palpur Wildlife Sanctuary, MNP – Madhav National Park, BTR – Bandhavgarh Tiger Reserve, PTR – Pench Tiger Reserve, PATR – Panna Tiger Reserve. Purple colour indicates tiger reserve; green - dense forest; light green - less dense forest; yellow - degraded forest.

One such protected area, Ranthambore Tiger Reserve (RTR), in Northwest India, was recognized as globally important for biodiversity conservation [6]. This was in spite of its isolation from other habitat blocks with tigers, fragmentation and high poaching pressures. RTR has substantial human populations within its boundaries whose agricultural, livestock farming and forest by-products collection activities bring villagers into regular and frequent competition and conflict with the needs of wildlife and conservation [4]. Despite years of ecodevelopment efforts, RTR is a wilderness island in a densely populated landscape of rural poor. However, this forest harbours a healthy albeit small population of tigers vital in national strategies for tiger conservation. Adequate protection and better management have so far ensured the survival of tigers in this reserve. There are a few anecdotal reports that some of these animals are dispersing out into neighbouring forests, like Kuno-Palpur Wildlife Sanctuary (KPWLS) and Madhav National Park (MNP) through highly fragmented and human-populated areas. KPWLS is about 100 kms to the south-east of RTR in Madhya Pradesh (Figure 1). Kuno Wildlife Division, spread over an area of 1280 km^2^ with a core sanctuary area of 345 km^2^, has been identified and prepared as a second home of the Asiatic lion, after Gir National Park, India, indicating good prey availability [7]. A further 100 kms to the east is the 354 km^2^ MNP, which is rich in ungulates and avifauna. At a considerable risk of human conflict and poaching, tigers from RTR can move through degraded and fragmented forest patches and agricultural fields to reach either of these two forests.

Tigers are solitary felid and ranges over large areas in search of new territories. However, there is very little evidence on how tigers move or how far they disperse, especially through fragmented and disturbed landscapes. Smith [8] reported an average dispersal distance of 33 kms for males and about 10 kms for females in Chitwan, Nepal. There are studies which also report sub-adult transients occasionally traveling far greater distances of 100 kms or more [8], [9]. However both these studies were carried out in good tiger habitats without fragmentation or human disturbances. Tigers are often extremely difficult to track or enumerate due to their elusive nature. Indirect evidences such as prey kills, scrape marks and pugmarks (paw prints) may indicate presence of tiger, but cannot be used to estimate numbers or understand movement. Photographic capture-recapture methods have been very effective in assessing tiger population dynamics in high-density forests like Nagarhole, India [10]. However these methods have several drawbacks in low tiger density areas, fragmented landscapes and areas with high levels of human presence [11]. Given these constraints, non-invasive genetic sampling is a potent technique to understand the structure of threatened tiger populations in degraded habitats.

The importance of protecting corridors and surrounding landscapes in order to enable animal movement has been extensively studied and highlighted in other animal species [12]–[14]. The present study is the first attempt to establish tiger movement across a fragmented landscape based on non-invasively obtained genetic data. We have ascertained tiger presence in KPWLS and MNP, and established their genetic connectivity with the animals of RTR. Determining migratory contact between these subpopulations can highlight important corridors which exist and those which are lost, thereby indicating priority areas for conservation [15]. In this study, we describe how migratory contact between animals of RTR, KPWLS and MNP persists even today despite high levels of forest fragmentation. Technological advances have now made it possible to identify individuals through unique genotypes, and this genetic data can be used to understand relationships among fragmented populations. Here, we use non-invasively collected faecal samples as a source of DNA for generating multilocus nuclear DNA genotypes, which can be further used for determining population structure and migratory patterns of tigers in the Northwest India. We also compared the genetic data of tigers obtained from these forests with that of tigers of Pench and Bandhavgarh Tiger Reserves in Madhya Pradesh, two prime tiger reserves of the Central Indian landscape. Our findings will help in establishing the importance of corridors and protection of connected forests in the longterm survival of tigers in a reserve like RTR that is subject to tremendous human pressures. Instead of managing and protecting each of these three forests, RTR, KPWLS and MNP, located in a highly human-dominated landscape, as an individual, isolated entity, the future of the tiger here may be better secured by managing these forests as part of a greater landscape with good connectivity.

## Materials and Methods

### Study area and sample collection

Ranthambore Tiger Reserve (RTR), spread over an area of 1334 km^2^, is located in the North-western state of Rajasthan, India (Figure 1). This reserve includes Ranthambore National Park which is about 392 km^2^. The area receives an average annual rainfall of approximately 800 mm from June to September. RTR is predominantly a tropical thorny and dry deciduous forest. There are more than 300 villages within a 5 km radius of the park with more than 150,000 people and livestock [16], [17]. The park lies at the edge of a plateau, and is river-bound to the north by Banas and to the south by Chambal. Other than tiger (*Panthera tigris*), the park supports a diverse population of mammals including large carnivore species like leopard (*Panthera pardus*), sloth bear (*Melursus ursinus*), hyena (*Hyaena hyaena*) and dhole (*Cuon alpinus*). Principal wild prey species are chital (*Axis axis*), nilgai (*Boselephas tragocamelus*), sambar (*Rusa unicolor*) and wild boar (*Sus scrofa*).

KPWLS (345 km^2^) is located in Sheopur district in northwest Madhya Pradesh, India and is surrounded by a buffer area of 900 km^2^. The habitat and fauna of this forest are similar to that in RTR. Sparsely-populated ravines between these two forests are probably used by dispersing tigers.

MNP is located in Shivpuri district in northwest Madhya Pradesh, India. It has a total area of 354 km^2^ and is predominantly a dry deciduous forest with sizeable lakes surrounded by grasslands. This forest is rich in avifauna and is winter home for several migratory birds. Predominant animal species within this park are chital, chinkara (*Gazella bennettii*), nilgai, sambar, blackbuck (*Antilope cervicapra*), common langur (*Semnopithecus entellus*), chowsingha (*Tetracerus quadricornis*), sloth bear and leopard. The lakes are habitat to marsh crocodile or mugger (*Crocodylus palustris*).

Between October and December 2010, fresh faecal samples were collected along all roads and trails within the core area of RTR and the adjoining buffer area. Samples were collected in two sampling occasions in the core area (392 km^2^) with a gap of twenty days to allow for the deposition of fresh samples. The buffer area (942 km^2^) was searched once for faecal samples. Fresh carnivore faecal samples were opportunistically collected by Forest Department personnel of MNP over a year (2010–2011) and sent to the Centre for Cellular and Molecular Biology (CCMB), Hyderabad, India for analysis. One fresh faecal sample found in KPWLS in April 2011 was similarly sent to the CCMB. Samples collected from Bandhavgarh tiger reserve (BTR) and Pench tiger reserve (PTR), Madhya Pradesh as part of a large-scale tiger monitoring program were also included in subsequent analyses. All samples, except the ones from RTR, were collected in fresh, self-adhesive plastic bags (Ziploc covers) with silica beads with their geographical locations appropriately recorded. RTR samples were preserved by the two-step method i.e. 24-hour storage in ethanol followed by desiccation with silica [18]. Once they reached the laboratory, all samples were stored at −20°C till further analysis. Permission to collect tiger scat samples in RTR was granted by Principal Chief Conservator of Forests (Wildlife) and Chief Wildlife Warden, Govt. of Rajasthan (letter no. 5252, dated 17^th^ May 2010). Samples from MNP were collected by forest officials and sent to the CCMB by the Field Director at different time points between 18^th^ February 2010 and 23^rd^ May 2011.The single sample from KPWLS was also collected by forest officials and sent to the CCMB by the Deputy Conservator of Forest (letter no. 2278, dated 30^th^ May 2011).

### DNA analysis

DNA was extracted from visibly fresh faecal samples by guanidinium thiocyanate-silica method [19] with minor modifications. This method gives results comparable to QIAamp DNA stool kit (Qiagen) in tigers [20], and has been extensively used in our studies. DNA was not isolated from crumbly or powdery samples, or samples with fungal growth. All isolations were carried out in a dedicated facility free from PCR products. Samples were extracted in sets of ten, which also included an extraction control to monitor for contamination at the time of isolation. All extracts were screened by a tiger-specific PCR assay [20] and only tiger-positive samples were further analyzed. Since faecal samples yield unpredictable amounts of low quality DNA, which can lead to subsequent genotyping errors, we quantified the amount of DNA in each tiger-positive sample by real-time PCR [21]. Samples which yielded sufficient quantities of usable DNA [21] were genotyped at twelve polymorphic microsatellite loci (F37, F42, F53, F96, F115, F124, F141, Fca391, Fca424, Fca441 [22]; and E6, E7; [20]). We followed the two-step multiplex PCR assay described by Arandjelovic *et al.* [23], with modifications. In the initial step, all 12 microsatellite loci were amplified together in a single reaction in triplicates. The PCR mixture (15 µl) consisted of 1XPCR Buffer (*TaKaRa ExTaq* Hot Start version, TaKaRa), 15 pM of each primer (unlabelled), 250 µM dNTPs, 1X BSA (New England Biolabs), 2 U of *Taq* enzyme (*TaKaRa ExTaq* Hot Start version, TaKaRa) and 5 µl of template DNA. PCR reactions were carried out in a Mastercycler epgradientS (Eppendorf) with the following conditions: initial denaturation at 95°C for 10 minutes, 40 cycles of 94°C for 15 seconds, 52°C for 20 seconds, 72°C for 30 seconds, followed by a final extension of 72°C for 30 minutes. Triplicate singleplex PCRs at each locus were carried out as above in reaction volumes of 15 µl, except that 0.5–0.7 µl of multiplex PCR product was used as template. PCR mix also contained 5 pM each of FAM or HEX fluorescently-labelled forward primer and unlabelled reverse primer. Cycling conditions were also similar as above except that primer-specific annealing temperatures for each singleplex PCR, varied from 50°C to 62°C. All PCR steps, except the addition of template DNA, were performed in a hood that was UV-irradiated before and after use to avoid contamination. PCR products from the singleplex amplification step were electrophoresed on an ABI 3730 Genetic Analyser and alleles were sized relative to an internal control (500 LIZ™, Applied Biosystems) using GeneMapper software version 3.7 (Applied Biosystems). Sex of putative individuals was determined by typing the zinc finger locus [24].

All allelic data were analyzed in Microsoft EXCEL spreadsheets. Allele frequency analysis, estimates of probability of identity (P_ID_) and P_ID_ (sib) were carried out in CERVUS version 3.0 [25], [26]. Unique genotypes were identified by the Identity Test in CERVUS. Samples which matched at a minimum of eight loci were pooled to create consensus genotypes, and samples which had mismatches at up to four loci were re-examined for possible genotyping errors. Allele frequencies were calculated in CERVUS, while private alleles were identified manually.

Tests for pairwise linkage disequilibrium among the microsatellite loci were done using FSTAT 2.9.3 [27]. Various parameters of population structure (F-statistics) were determined as described by Weir and Cockerham [28]. Jackknifing procedure was applied over loci to derive significance levels and bootstrapping was done to derive 95% confidence intervals for these statistics. Parameters of population structure are defined as the correlations between pairs of genes (i) within individuals (F) (ii) between individuals in the same population (θ), and (iii) within individuals within populations (f), and are analogous to Wright's [29] F_IT_, F_ST_ and F_IS_, respectively.

### Population structure

We first tried to ascertain patterns of variations in the sampled tiger populations in Central India by Principal Coordinate Analysis (PCA). This is a multivariate technique that allows one to find and plot the major patterns within a multivariate data set (e.g., multiple loci and multiple samples). PCA was done using GenAlEx 6.1 [30], where the procedure is based on an algorithm published by Orloci [31].

We used two different Bayesian analyses to understand the structure in Central Indian tiger populations investigated in this study. First, we used the model-based clustering method in STRUCTURE 2.3.2 [32] to determine optimal number of genetic clusters (*K*) without any prior population assignment. In this method the program calculates fractional membership of each individual in each cluster (*Q*). The most appropriate K value was obtained based on the method described by Evanno *et al.*, [33]. Analysis was performed at least five times using more than 70,000 replicates and 30,000 burn-in cycles under the admixture model.

Next, we performed an exclusion test [34] in GENECLASS 2.0. Using the simulation method by Paetkau *et al.* [35], we tried to test whether each individual tiger actually originated from the sampled areas. The probability of individual genotypes coming from each sampled locality was calculated by comparing individual genotypes to 10000 simulated individuals per locality [15].

### Detection of migrants

STRUCTURE 2.3.2 and GENECLASS 2.0 were also used to identify first-generation migrants and individuals with mixed ancestry. In this case, prior population information was used in the USEPOPINFO option in STRUCTURE to determine the individuals that were not residents of their sampled population. STRUCTURE cluster membership inferred from the above clustering analysis was used as prior population information for this test. As we have no information about migration, migration rate (MIGPRIOR) was assigned as an initial condition [36]. Number of burn-ins and total number of replicates were the same as in the previous analysis without prior population information.

We selected the ‘detect migrants’ function in GENECLASS 2.0 as it is explicitly designed to identify first generation migrants [37] i.e. individuals born in a population other than the one in which they were sampled [15]. We used the L*_h_*/L*_max_* likelihood test statistics to identify migrants. We used the Bayesian criterion of Rannala and Mountain [38] in combination with the resampling method of Paetkau *et al.* [35], to determine the critical value of L*_h_*/L*_max_* beyond which individuals were assumed to be migrants. We selected an alpha level of 0.05 to determine critical values [35].

## Results

### Individual identification and data analysis

Out of the 221 faecal samples collected from RTR between October and December 2010, 198 were found suitable for DNA isolation. Difficult terrain and bad weather prevented the collection of consistently high quality samples from all areas. Of the 115 tiger positive samples, real time quantification revealed that 82 (71.3%) contained sufficient nuclear DNA for subsequent genotyping. The single sample from KPWLS was of tiger origin and yielded good DNA. Seventeen faecal samples were received from MNP between February 2010 and May 2011. Eight of these samples were found to be of tiger origin, six of which yielded sufficient amounts of nuclear DNA. Out of the total set of genotypes from RTR, we selected eleven unique individuals, four males and seven females, from different locations within RTR so as to get a fair representation of the entire population. Six DNA extracts from MNP yielded six distinct genotypes, three males and three females; while the KPWLS sample was from a male tiger. Genotype data of ten individuals from BTR and fifteen from PTR, Madhya Pradesh were also included in the current investigation (Table 1). Mean expected heterozygosity over twelve loci used for RTR, KPWLS and MNP genotypes was 0.6961, while observed heterozygosity for the same samples was 0.7624. Individual probability of identity for the twelve polymorphic microsatellite loci used in this study was 1.28E-0010 at the third locus, while sibling probability of identity was 6.664×10^−5^ at the sixth locus making it very unlikely that two individuals would have identical genotypes. While calculating allelic richness, we included the sample from KPWLS in the MNP population. Allelic richness describes the number of alleles per locus independent of sample size and its values ranged from 3 to 8 (Table 2). We also attempted to identify private alleles and a majority of these were found in the BTR population (10), followed by PTR (5), RTR (3) and MNP (1) (Table 3).

**Table 1:** Extraction of DNA, genotyping and sexing of samples based on tiger scats collected from Ranthambore, Kuno-Palpur, Madhav, Bandhavgarh and Pench Tiger Reserves.

Forest	Faecal samples collected	Samples used for DNA isolation	Tiger positive samples	Samples with amplifiable amounts of nuclear DNA	Individuals used in the study	Males	Females
Ranthambore (RTR)	221	198	115	82	11	4	7
Kuno-Palpur (KPWLS)	1	1	1	1	1	1	
Madhav (MNP)	17	17	8	6	6	3	3
Bandhavgarh (BTR)	217	208	161	136	10	7	3
Pench (PTR)	306	304	104	94	15	4	11

**Table 2:** Number of alleles per locus in different populations studied (Allelic Richness).

Locus	PTR	BTR	RTR	MNP
F37	4	—	4	4
F42	5	5	5	5
F53	7	5	5	4
F115	4	4	3	3
F124	5	8	5	3
F141	5	5	4	3
Fca391	3	3	4	4
Fca424	5	4	7	4
Fca441	4	4	4	4
F96	6	3	4	4
E6	7	4	6	4
E7	5	3	4	4

**Table 3:** Private alleles in different tiger populations of the Central Indian Landscape.

Forest	Locus	Allele	Frequency
PTR	F96	175	0.197
		179	0.332
		185	0.040
	F53	178	0.023
		188	0.046
BTR	F391	222	0.809
	F124	200	0.104
		224	0.051
		228	0.105
	F53	184	0.051
	F115	175	0.200
		191	0.278
		195	0.222
	E7	151	0.105
		153	0.345
RTR	F424	174	0.094
	E6	138	0.146
		159	0.046
MNP	F42	234	0.143

Overall mean for Wright's F-statistics [28] of the RTR and MNP populations was significantly different from zero. Relatedness among individuals in the given dataset was also significantly different from zero. Overall Rst, an estimator of genetic differentiation among these samples, was 0.011 and θ (Fst) was 0.041, respectively (Table 4) indicating a diverse genetic population and lack of inbreeding. The two populations did not show significant linkage disequilibrium (P- value for 0.05% was <0.05). All f (F_IS_) estimates across the loci showed heterozygote excess based on table wide randomizations (P<0.05). Overall averaged f estimates ranged from −0.041 to −0.297 with an average of −0.122±0.049 for these two populations. FIT estimates (−0.077) revealed that the populations are in Hardy–Weinberg equilibrium.

**Table 4:** Wright's F-statistics analysis for Madhav National Park and Ranthambore Tiger reserve populations.

*Loci*	*f(F_IS_)*	*θ (F_ST_)*	*F (F_IT_)*	*Relat*	*Relatc*	*R_st_*
Over all	−0.122	0.041	−0.077	0.089	0.112	0.011
SE^a^	0.049	±0.027	±0.043	0.058		

F_IS_, F_ST_, and F_IT_ are correlations between pairs of genes, within individuals within populations, between individuals in the same population and within individuals, respectively.

Relat, an estimator of the average relatedness of individuals within samples when compared to whole [59].

Relatc estimates the inbreeding corrected relatedness [60].

R_st_, estimate of relative genetic differentiation.

a Standard errors – estimate from jackknife over loci and significance from t-test using these estimates, p<0.05.

### Population structure

**Figure 2:**
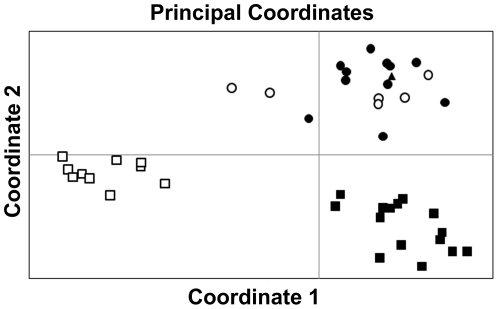
Principal Coordinate Analysis of genotypes obtained from “O” Madhav National Park (MNP), “▴” Kuno-Palpur Wildlife Sanctuary (KPWLS), “•” Ranthambore Tiger Reserve (RTR), “▪” Pench Tiger Reserve (PTR) and “□” Bandhavgarh Tiger Reserve (BTR) genotypes.

For all subsequent population genetic analyses, we used the genotype data of the 43 distinct individuals described above. This was done in order to compare the two new populations (KPWLS and MNP) with tigers from the three well established, but geographically distinct populations (RTR, BTR and PTR) in Central India. Principal Coordinate Analysis (PCA) of these populations in GenAlEx 6.1 shows that animals from PTR, BTR and RTR form distinct clusters. All individuals from MNP and KPWLS cluster closely with RTR animals, although a few from MNP appear to be distinct and not part of the RTR cluster (Figure 2).

**Figure 3:**
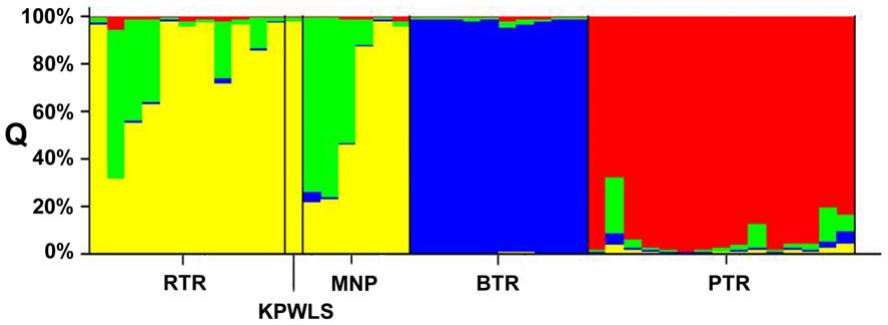
Proportional membership of each tiger in the four clusters identified by STRUCTURE. Each tiger is represented by a single vertical bar. RTR – Ranthambore Tiger Reserve, KPWLS – Kuno-Palpur Wildlife Sanctuary, MNP – Madhav National Park, BTR – Bandhavgarh Tiger Reserve, PTR – Pench Tiger Reserve, Madhya Pradesh.

The dataset was examined using STRUCTURE under different assumptions of number of population clusters (k = 1, k = 2……..k = 10) without any pre-assignment of population affiliation. Calculation of Δ*K* from the output, as described by Evanno *et al.*, [33], produced a modal value of the statistic at *K* = 4, followed by a second mode at *K* = 5. Although there is evidence for population substructuring at both *K* = 4 and *K* = 5, *K* = 4 appears optimal as it is the lowest value [15], [35], [36]. All analyses showed consistent and identical clustering of MNP and KPWLS populations with RTR animals, and these are distinctly different from BTR and PTR populations (Figure 3). The single KPWLS individual has full ancestry in RTR cluster (*Q* = 0.01). MNP is made up of two clusters with half the individuals belonging to either cluster. Three of the MNP tigers show full membership to the RTR cluster (mean *Q* = 0.046, range 0.007–0.11). The remaining three tigers show partial RTR ancestry (mean *Q* = 0.67, range 0.52–0.76), and could have partial genetic ancestry with MNP or some other tiger areas. Similarly four RTR individuals show partial ancestry (mean *Q* = 0.55, range 0.31–0.72), while the remaining seven show full membership to RTR cluster (mean *Q* = 0.95, range 0.86–0.98).

The rate at which individuals are correctly assigned to their sampled locality can also be used as an assessment of population genetic structure [15], [39]. Population assignment test using GENECLASS 2.0 accurately assigned 39 (90.7%) of the 43 individuals to their respective populations. The four misassigned individuals were later identified as migrants from RTR. All individuals from BTR and PTR were correctly assigned to their respective forests.

### Detection of migrants and admixed individuals

Both STRUCTURE and GENECLASS detected the same individuals as migrants (Table 4) in the RTR-KPWLS-MNP group. STRUCTURE identified four individuals (KPWLS1, MNP4, MNP5 and MNP6) as migrants from RTR to KPWLS and MNP (*P* = 0.978, 0.938, 0.983, 0.964). GENECLASS identified the same individuals as first generation migrants with the L*_h_*/L*_max_* ratio (Table 5). STRUCTURE also identified a few individuals that were neither readily classified as migrants nor as residents, suggesting that these animals may be of admixed ancestry. These individuals have Q-values between 0.2 and 0.8 [15], [40], [41]. Seven individuals of mixed ancestry were identified in the same RTR-KPWLS-MNP group, four from RTR (RTR2, RTR3, RTR4 and RTR8) and three from MNP (MNP1, MNP2, MNP3). GENECLASS was not as efficient as STRUCTURE in identifying admixed individuals and thirteen individuals from RTR and MNP had low or similar assignment probabilities to both localities. These included the seven individuals identified by STRUCTURE as having mixed ancestry.

**Table 5:** Detection of migrant tigers in the Northwest India.

SN	Sample	Geographic origin	Structure Q (PTR/MNP/BTR/RTR clusters; no prior population information, K = 4)	Geneclass locality of highest probability assignment–exclusion test	Geneclass highestassignment probability	Geneclass *F*_0_migrantlikelihood ratio(*L_h_/L_max_*)*P<0.05	Structuremigrantprobability	Final migrant/admixture/residentclassification
1	RTR-1	Ranthambore	0.005/0.019/0.005/0.972	RTR	0.8871	0.000	0.010	RD
2	RTR-2	Ranthambore	0.051/0.628/0.006/0.315	RTR	0.0330	0.000	0.386	AD
3	RTR-3	Ranthambore	0.008/0.434/0.006/0.552	RTR	0.1868	0.000	0.219	AD
4	RTR-4	Ranthambore	0.012/0.350/0.007/0.631	RTR	0.3906	0.000	0.159	AD
5	RTR-5	Ranthambore	0.005/0.007/0.003/0.985	RTR	0.1638	0.000	0.005	RD
6	RTR-6	Ranthambore	0.019/0.020/0.003/0.958	RTR	0.0629/0.0908	0.000	0.020	RD
7	RTR-7	Ranthambore	0.008/0.015/0.003/0.974	RTR	0.8891	0.000	0.011	RD
8	RTR-8	Ranthambore	0.017/0.244/0.020/0.719	RTR	0.1159	0.000	0.170	AD
9	RTR-9	Ranthambore	0.011/0.019/0.004/0.966	RTR	0.4046	0.000	0.026	RD
10	RTR-10	Ranthambore	0.006/0.128/0.005/0.861	RTR	0.1009/0.1008	0.000	0.075	RD
11	RTR-11	Ranthambore	0.005/0.013/0.004/0.978	RTR	0.7822	0.000	0.008	RD
12	KPWLS-1	Kuno-Palpur	0.006/0.010/0.005/0.978	RTR	0.7942	3.668*	0.978	MS
13	MNP-1	Madhav	0.004/0.738/0.039/0.219	MNP	0.0050	0.000	0.381	AD
14	MNP-2	Madhav	0.004/0.760/0.006/0.229	MNP	0.0090	0.000	0.403	AD
15	MNP-3	Madhav	0.012/0.520/0.004/0.465	MNP	0.1499/0.3363	0.000	0.255	AD
16	MNP-4	Madhav	0.010/0.111/0.005/0.873	RTR	0.3906	0.028*	0.938	MS
17	MNP-5	Madhav	0.006/0.007/0.003/0.983	RTR	0.7722	0.327*	0.983	MS
18	MNP-6	Madhav	0.017/0.020/0.003/0.960	RTR	0.3157	0.046*	0.964	MS

MS, migrant whose source locality was determined; AD, admixed individual; RD, resident.

## Discussion

By using non-invasively collected genetic data, we could determine tiger presence in MNP, and also establish relatedness of these animals with tigers of RTR, thereby establishing that tigers move between these two protected areas most probably via KPWLS. The microsatellite markers selected in this study are informative enough to identify genetic diversity, migration and population structure within closely related populations. Further, the numbers of individuals analyzed represent approximately 40 (RTR, BTR and PTR) to 100% (KPWLS and MNP) of existing tiger populations in these protected areas [42]. Previous surveys based on indirect evidences reported possible presence of three tigers in approximately 3000 km^2^ landscape which includes KPWLS and MNP [42]. We identified, both by STRUCTURE and GENECLASS, four tigers in the given dataset which have migrated out from RTR in this generation to KPWLS and MNP (Table 5). However, there is no evidence of first generation migration in the opposite direction. This may be because RTR has reached its full carrying capacity and young animals are forced to move out in search of new territories. Sub-adult tigers are known to move over long distances to establish their own territories [10], [43]. This movement of tigers out of RTR is however not a new phenomenon as there are tigers in MNP with mixed RTR ancestry (Figure 3; Table 5). The most interesting finding in this study is the presence of admixed individuals in RTR with MNP or possibly a different ancestry which is also evident in MNP tigers. The presence of such admixed individuals suggests that these tigers have not only moved over long distances between forests but have also been able to reproduce in new areas, thereby contributing to the genetic diversity of subpopulations. Such dispersal and subsequent reproduction is crucial for the maintenance of long-term genetic health in small fragmented populations [15]. This finding highlights the healthy connectivity which existed between RTR and MNP and which is progressively getting fragmented [42].

RTR in India is located in an extremely tiger-hostile landscape. Substantial efforts to manage and protect this reserve have ensured that tigers persist here today, but with increased risk of tiger-human conflict which can severely hamper conservation efforts. Further, RTR needs well-protected dispersal corridors to other forests to ensure tiger movements in the greater landscape and to prevent loss of genetic diversity with subsequent inbreeding within tigers of this forest. KPWLS and MNP are located reasonably close to RTR (Figure 1), but have also been shown to be extremely sensitive to hostility of the surrounding landscape matrix [5]. All three protected areas mentioned above are part of a Level III Tiger Conservation Unit (TCU), indicating that this landscape has low probability of long-term persistence of tiger populations due to various reasons such as small size, isolation from other tiger habitats, fragmentation and high poaching pressures. But these forests are extremely important to national conservation strategies and, with intensive management and protection, can harbour small tiger populations [44].

There is a strong possibility that we have not sampled at least one tiger population in the Central Indian landscape which might have contributed significantly to the genetic structure of MNP population and consequently to the admixed individuals in RTR. There probably existed historic movements of tigers from other locations such as Chambal ravines, Panna Tiger Reserve and BTR into MNP; however, this link is apparently lost now [42]. Tigers of Panna Tiger Reserve were all lost to poaching in the last decade and therefore could not be included in this analysis. However, there is a possibility that some of the individuals in MNP have migrant ancestry of those populations, which further migrated to and mixed with tigers of RTR. This study indicates that RTR and MNP tiger populations have good genetic diversities (Table 4), and there still exists first generation migration at least from RTR towards MNP. As mentioned earlier, this may be forced migration of young animals risking their lives through hostile terrains to reach new territories, and may represent a small fraction of animals which actually attempted moving through this landscape. DeFries *et al.* [45], reported loss of nearly 70% of the surrounding buffers during the last 20 years, especially in dry tropical forests of South and Southeast Asia. If this trend continues at the present rate, tigers will no longer be able to move between protected areas, leading to cannibalism, inbreeding depression and local extinction [46]–[50], provided other stochastic factors do not eliminate them first [51]. Further, RTR and MNP together have lesser number of private alleles in the twelve loci used (Table 3), compared to the better tiger habitats, PTR and BTR. However these alleles are unique to the fragile RTR population, and MNP contributes substantially (25%) to this uniqueness.

Despite major conservation initiatives, the last ten to fifteen years have witnessed more than 40% decline in the estimated area known to be occupied by tigers [52], and the current global tiger range is only 7% of its historic range. Ranganathan *et al.* [5], developed a landscape scale, density-based model to determine which areas and management practices are suitable for future survival of tigers in the Indian subcontinent. Their study indicates that the subcontinent can potentially hold 3500–6500 tigers in about 150 reserves, but just 21 of these reserves can hold most (58–95%) of this tiger capacity. These high population target reserves are relatively insensitive to the hostility of the surrounding landscape matrix. Efficient management of these reserves irrespective of the surrounding landscape will help in improving tiger numbers. However this is not the case in the remaining 129 reserves (85% of the total) which are highly sensitive to surrounding pressures, and are to be unable to sustain populations within a tiger-hostile matrix, even with reasonable management. Tigers in these protected areas can only persist as part of larger populations that extend into surrounding forests. The authors further suggested that conservation of tigers in these areas requires joint management of protected areas and the greater landscapes [5].

Understanding population structure and connectivity is crucial for determining units of management for wildlife conservation programmes [53]–[58]. Population structure and migration detected in RTR and MNP tigers have important implications for protection and management of this charismatic species in Northwest India. We propose that substantial conservation efforts must focus on maintenance and improvement of connectivity between RTR, KPWLS and MNP. Since these forests are located in different states (Madhya Pradesh and Rajasthan) of India, collaborative efforts should be made to protect this trans-boundary landscape. Forests in this landscape already carry different protection status, but the corridors in between them are given the least conservation priority and are vulnerable to human activities. As these forests are located within a human-dominated, tiger-hostile landscape, it is very important that the corridors between the forests are better protected so as to ensure tiger movements and longterm survival of tigers in this landscape. Efforts should also be made to restore the corridor between MNP and Panna Tiger Reserve in Central India. Our study has also highlighted the potential of Madhav National Park to sustain breeding populations of tigers; it therefore, deserves the status of a Tiger Reserve, which would ensure better management and protection. Kuno-Palpur Wildlife Sanctuary is also a suitable habitat with good prey abundance, and should be surveyed extensively for tiger presence and abundance.
